# Mapping the tRNA modification landscape of *Bartonella henselae* Houston I and *Bartonella quintana* Toulouse

**DOI:** 10.3389/fmicb.2024.1369018

**Published:** 2024-03-13

**Authors:** Samia Quaiyum, Jingjing Sun, Virginie Marchand, Guangxin Sun, Colbie J. Reed, Yuri Motorin, Peter C. Dedon, Michael F. Minnick, Valérie de Crécy-Lagard

**Affiliations:** ^1^Department of Microbiology and Cell Science, University of Florida, Gainesville, FL, United States; ^2^Department of Biological Engineering, Massachusetts Institute of Technology, Cambridge, MA, United States; ^3^Singapore-MIT Alliance for Research and Technology, Singapore, Singapore; ^4^Université de Lorraine, UAR2008/US40 IBSLor, EpiRNA-Seq Core Facility and UMR7365 IMoPA, CNRS-Inserm, Biopôle UL, Nancy, France; ^5^Division of Biological Sciences, University of Montana, Missoula, MT, United States; ^6^Genetic Institute, University of Florida, Gainesville, FL, United States

**Keywords:** intracellular pathogens, tRNA modification, mass spectrometry, next-generation sequencing, Bartonella

## Abstract

Transfer RNA (tRNA) modifications play a crucial role in maintaining translational fidelity and efficiency, and they may function as regulatory elements in stress response and virulence. Despite their pivotal roles, a comprehensive mapping of tRNA modifications and their associated synthesis genes is still limited, with a predominant focus on free-living bacteria. In this study, we employed a multidisciplinary approach, incorporating comparative genomics, mass spectrometry, and next-generation sequencing, to predict the set of tRNA modification genes responsible for tRNA maturation in two intracellular pathogens—*Bartonella henselae* Houston I and *Bartonella quintana* Toulouse, which are causative agents of cat-scratch disease and trench fever, respectively. This analysis presented challenges, particularly because of host RNA contamination, which served as a potential source of error. However, our approach predicted 26 genes responsible for synthesizing 23 distinct tRNA modifications in *B. henselae* and 22 genes associated with 23 modifications in *B. quintana*. Notably, akin to other intracellular and symbiotic bacteria, both Bartonella species have undergone substantial reductions in tRNA modification genes, mostly by simplifying the hypermodifications present at positions 34 and 37. *Bartonella quintana* exhibited the additional loss of four modifications and these were linked to examples of gene decay, providing snapshots of reductive evolution.

## 1 Introduction

The conversion of genetic information from messenger RNA (mRNA) into functional proteins represents a fundamental and intricately regulated process in all living organisms (Zaher and Green, 2009). At the heart of this complex mechanism, transfer RNA (tRNA) molecules function as indispensable adaptors, ensuring the precise and faithful translation of mRNA codons into amino acids (Berg and Brandl, 2021). Post-transcriptional modifications of tRNA molecules play pivotal roles in preserving the stability and integrity of tRNA molecules while enhancing the accuracy and efficiency of the decoding process (El Yacoubi et al., 2012). As tRNA modification levels can be affected by stress or the metabolic status of cells, and these variations can affect the translation of specific mRNAs, specific modifications can be recruited to fulfill regulatory roles (Chan et al., 2010; Helm and Alfonzo, 2014; de Crécy-Lagard and Jaroch, 2021; Wang and Lin, 2023). Even though such regulatory roles were postulated over 40 years ago (Persson, 1993), it is only recently that the full regulatory or homeostatic loops have been dissected in different model organisms, including several pathogenic bacteria where they have been found to have roles in resistance to stress and virulence (Koh and Sarin, 2018; Antoine et al., 2021; de Crécy-Lagard and Jaroch, 2021; Fruchard et al., 2022).

Exploration of the intricate regulatory mechanisms driven by tRNA modifications necessitates a comprehensive understanding of the entire set of modifications and the corresponding genes encoding the enzymes responsible for their installation. However, this knowledge remains largely incomplete, with significant gaps existing beyond a few well-studied model organisms, including the bacterial pathogen *Mycoplasma capricolum* (de Crécy-Lagard and Jaroch, 2021). Advancing our understanding requires the integration of analytical tools such as mass spectrometry (MS) or next-generation sequencing with comparative genomic analyses across diverse organisms spanning the tree of life (de Crécy-Lagard and Jaroch, 2021). Recent years have seen integrated studies that successfully cataloged tRNA modifications and associated genes in a few pathogenic bacteria, such as *Vibrio cholerae* (Kimura et al., 2020) and *Mycobacterium tuberculosis* (Tomasi et al., 2023), as well as in the antibiotic-producing *Streptomyces albidoflavus* (Koshla et al., 2023). Notably absent from these investigations are members of obligate intracellular bacterial families like *Chlamydiaceae*, *Rickettsiaceae*, or *Ehrlichiaceae* which harbor significant pathogenic species for humans (McClure et al., 2017; Loterio et al., 2021). Detecting tRNA modifications in intracellular pathogens presents unique challenges, including the low yield of tRNA during preparation and the potential for contamination by host cell tRNA. Yet, beyond unraveling the potential roles of tRNA modifications in the virulence of intracellular bacteria, mapping these modifications can contribute to our understanding of the genetic code’s evolution in organisms that undergo extensive genome reductions as part of their adaptations to specialized niches (Andersson and Kurland, 1998; Albalat and Cañestro, 2016).

*Bartonella henselae* Houston I, a member of the Hyphomicrobiales order, is a facultative intracellular Gram-negative pathogen that induces a spectrum of diseases, varying in severity. *Bartonella henselae* is linked to ailments such as cat-scratch disease, which primarily affects children, and bacillary angiomatosis (BA), more commonly observed in HIV/AIDS patients (Rovid Spickler, 2005; Raoult, 2007). Additionally, *Bartonella quintana* Toulouse, another facultative intracellular pathogen transmitted by human body lice, leads to trench fever and related illnesses (Alsmark et al., 2004; Foucault et al., 2006). These pathogens have complex life cycles as their reservoirs are mammalian vascular cells, but they are transmitted by arthropod vectors. *Bartonella henselae* has evolved from an insect symbiont ancestor undergoing a first cycle of genome reduction (Segers et al., 2017) with *B. quintana* undergoing a subsequent massive secondary genome reduction (Engel and Dehio, 2009). In addition, as these organisms can be readily cultured *in vitro* with vascular cells (erythrocytes, endothelial cells; Battisti and Minnick, 2008), they can be used as valuable models for employing the MS/NGS/comparative genomic approach to identify all genes related to tRNA modifications in intracellular bacteria.

Prior studies, which concentrated on rescuing the wobble base modification Queuosine (Q) in *B. henselae* and *B. quintana*, revealed that while the latter bacterium lost all genes associated with the pathway, *B. henselae* retained only two out of eight Q synthesis/salvage genes. These two genes constitute a basic salvage pathway, comprising a QPTR/YhhQ family transporter and a homolog of tRNA-guanosine (34) preQ_1_ transglycosylase (Tgt; Quaiyum et al., 2023). Interestingly, this minimal pathway not only successfully salvages the Q precursor PreQ_1_ but also enables the rescue of the q base when present in high concentrations, a capability not shared by the *E. coli* orthologs. It is not clear which conditions would allow the salvage of these two precursors in natural environments, but analysis of tRNA extracted from *B. henselae* cells showed that preQ_1_ could be detected at the wobble position, a unique situation, to date. The current study focuses on predicting the remaining tRNA modifications to generate a better picture of decoding in these organisms.

## 2 Methods

### 2.1 Bioinformatic analyses

The genomic sequences of *Bartonella henselae* str. Houston I (NCBI Taxon ID 38323; NC_005956.1) and *Bartonella quintana* str. Toulouse (NCBI Taxon ID 283165; NC_005955) were used for all analyses. tRNA isoforms information was obtained from GtRNA database (Chan and Lowe, 2016).^1^,^2^ The BV-BRC platform (Olson et al., 2023) was used for protein family identifications, physical clustering analyses, and species tree construction using FastTree (version 47). Species trees were also generated using PhyloT^3^ (database version 2023.2) and Protein families were also identified with KO numbers in the KEGG database (Kanehisa et al., 2021). UniProt was used for ID mapping and advanced search tools (Bateman et al., 2023). NCBI was used for BlastP analyses (Altschul et al., 1997) and literature searches (NCBI Resource Coordinators, 2016). GizmoGene^4^ was used to make the physical clustering figures and Modomics (Boccaletto et al., 2018) for analyses of tRNA sequences. iToL^5^ (version 6.8.1; Letunic and Bork, 2021) and MORPHEUS^6^ were used to visualize the presence/absence data of protein families along the species trees.

### 2.2 Strains, media and bulk tRNA preparation

*Bartonella henselae* Houston I was obtained from the American Type Culture Collection (ATCC 49882). *Bartonella quintana* Toulouse was a generous gift from Volkhard Kempf (Goethe-Universität Frankfurt). Both species were cultivated as previously described (Battisti and Minnick, 2008) on HIBB agar plates [i.e., Bacto heart infusion agar (Becton, Dickinson, Sparks, MD) supplemented with 4% defibrinated sheep blood and 2% sheep serum (Quad Five, Ryegate, MT) by volume] for 4 days (*B. henselae*) or 10 days (*B. quintana*) at 37°C, 5% CO_2_ and 100% relative humidity. Bacteria in free form were harvested into ice-cold heart infusion broth from the surface of HIBB plates using a sterile razor blade as previously described (Battisti and Minnick, 2008). This method left sheep cells embedded in the agar matrix and minimized the chance of cross-contamination. The bacterial suspension was centrifuged for 2 min at 16,000 × g at 4°C, and the resulting pellet was suspended in 1 mL of Trizol (Thermo Fisher Scientific, Waltham, MA, United States). Small RNAs were extracted with a PureLinkTm miRNA Isolation kit following manufacturer’s instructions (Thermo Fisher Scientific). Purified RNAs were eluted using 50 μL of RNase-free water and quantified using a Nanodrop 1000 spectrophotometer (Quaiyum et al., 2023).

### 2.3 LC-MS analysis of nucleosides in tRNA samples

tRNA modifications were analyzed by LC-MS using two similar methods. For samples S1–S3 in Supplementary Tables S1–S3, 3 μg of small RNA was hydrolyzed in a 50 μL (0.06 μg/μL) digestion cocktail containing 12.5 U of U benzonase (0.25 U/μL), 5 U CIAP (calf intestinal alkaline phosphatase; 0.1 U/μL), 0.15 U of PDE I (phosphodiesterase I; 0.003 U/μL), 0.1 mM deferoxamine, 0.1 mM BHT (butylated hydroxytoluene), 5 ng coformycin (0.1 ng/μL), 50 nM ^15^N-dA (internal standard [^15^N]_5_-deoxyadenosine), 2.5 mM MgCl_2_ and 5 mM Tris–HCL buffer pH 8.0. For samples S1–S5 in Supplementary Tables S1, S4, S5, 1.8 μg of small RNA was hydrolyzed in 30 μL (0.06 μg /μL) digestion cocktail containing 2.49 U benzonase (0.083 U/μL), 3 U CIAP (0.1 U/μL), 0.07 U PDE I (0.002 U/μL), 0.1 mM deferoxamine, 0.1 mM BHT, 3 ng coformycin (0.1 ng/μL), 25 nM ^15^N-dA, 2.5 mM MgCl_2_ and 5 mM Tris–HCL buffer pH 8.0. The digestion mixture was incubated at 37°C for 6 h. After digestion, all samples were analyzed by chromatography-coupled triple-quadrupole mass spectrometry (LC-MS/MS). For each sample, hydrolysate containing 600 ng of RNA was injected for each of the technical replicates (Supplementary Tables S3, S5). Using synthetic standards, HPLC retention times of RNA modifications were confirmed on a Waters Acuity BEH C18 column (50 × 2.1 mm inner diameter, 1.7 μm particle size) coupled to an Agilent 1,290 HPLC system and an Agilent 6,495 triple-quadrupole mass spectrometer (Supplementary Table S1). All references for the synthesis of the standards have been included to Table 1. The Agilent sample vial insert was used. The HPLC system was operated at 25°C and a flow rate of 0.35 mL/min or 0.3 mL/min in a gradient [Supplementary Tables S2, S4 with Buffer A (0.02% formic acid in water)] and Buffer B (0.02% formic acid in 70% acetonitrile). The HPLC column was coupled to the mass spectrometer with an electrospray ionization source in positive mode with the following parameters: dry gas temperature, 200°C; gas flow, 11 L/min; nebulizer, 20 psi; sheath gas temperature, 300°C; sheath gas flow, 12 L/min; capillary voltage, 3,000 V; nozzle voltage, 0 V. Multiple reaction monitoring (MRM) mode was used for detection of product ions derived from the precursor ions for all the RNA modifications with instrument parameters including the optimized collision energy (CE) optimized for maximal sensitivity for the modification. Signal intensities for each ribonucleoside were normalized by dividing by the sum of the UV signal intensities of the four canonical ribonucleosides recorded with an in-line UV spectrophotometer at 260 nm. The MS data was deposited in the PRIDE database^7^ with the accession number PXD048805.

**Table 1:** Summary of ribonucleosides identified by LC-MS in *Bartonella henselae.*

Modification detected by MS	Ref^%^	Gene(s) linked	NGS data	Position	Location	Potential contamination
ac^4^C	van Montagu et al. (1968)	Yes	No	34	ASL	
A_m_	Hyde et al. (2003)	Yes	Yes	32		
C_m_	Nyilas et al. (1986)	Yes	Yes	multiple	ASL	
cmnm^5^s^2^U	Bartosik and Leszczynska (2015)	Yes	No	34	ASL	
D	Luvino et al. (2006)	Yes	No	multiple	Body	
Gm	Chow et al. (2003)	No	No	NA	NA	Yes
ho^5^U	Gavriliu et al. (2000)	yes	Yes	34	ASL	
I	Xu et al. (2021)	yes	No	34	ASL	
i^6^A	Corder et al. (2013)	yes	No	37	ASL	
m^1^A	Broom et al. (1964)	No	No	NA	NA	Yes
m^1^G	Höbartner et al. (2003)	Yes	No	37	ASL	
m^2^_2_G	Höbartner et al. (2003)	No	No	NA	NA	Yes
m^2^A	Van Aerschot et al. (1993)	Yes	No	NA	ASL	
m^2^G	Höbartner et al. (2003)	No	No	NA	NA	Yes
m^3^C	Sun and Singer (1974)	No	No	NA	NA	Yes
m^3^U	Höbartner et al. (2003)	No	No	NA	NA	Yes
m^5^C	Abdel Rahman et al. (2001)	No	No	NA	NA	Yes
m^5^U	Knoblauch (1999)	Yes	No	54	Body	
m^6^_6_A	Miles et al. (1995)	No	No	NA	NA	Yes
m^6^A	Höbartner et al. (2003)	Yes	No	37	ASL	
m^7^G	Pagano et al. (1995)	Yes	Yes	46	Body	
ms^2^i^6^A	Corder et al. (2013)	Yes	No	37	ASL	
ms^2^t^6^A*		Yes	No	37	ASL	
preQ_1_	Kondo et al. (1986)	Yes	No	34	ASL	
Q	Kondo et al. (1986)	Yes	No	34	ASL	Yes
s^2^C	Robin et al. (2006)	Yes	No	32	ASL	
s^2^U	Wang et al. (2017)	Yes	No	34	ASL	
t^6^A	Debiec and Sochacka (2021)	Yes	No	37	ASL	
U_m_	Nyilas et al. (1986)	Yes	Yes	32	ASL	
Ψ	Sato et al. (1984)	Yes	Yes	Multiple	ASL & Body	

^%^References for protocols used to synthesize the standards. ^*^No standard. Modifications predicted to be present in tRNAs with high confidence are bolded. ASL, Anticodon-Stem-Loop. NA, Not Applicable.

### 2.4 tRNA modification analysis by next generation sequencing

Analysis of tRNA modifications present in *B. henselae* tRNA fractions was performed by a combination of three previously published original protocols, namely RiboMethSeq, AlkAnilineSeq and HydraPsiSeq. Additional analysis of eventual m^5^C residues was done by standard RNA bisulfite sequencing. All sequencing data was deposited in the Short Read Archive^8^ with the accession number PRJEB72223.

#### 2.4.1 RiboMethSeq

RiboMethSeq protocol allowed us to map 2′-O-methylations by assessing their protection against alkaline cleavage (Marchand et al., 2016, 2017). To further enhance our analysis, we also extracted reverse transcriptase (RT) misincorporation signatures at RT-arresting nucleotides (Motorin and Marchand, 2018; Werner et al., 2020). As part of the RiboMethSeq tRNA analysis, total RNA from *B. henselae* was fragmented under alkaline conditions, followed by library preparation and sequencing (Marchand et al., 2017). The RT misincorporation signatures were derived from the Samtools mpileup format and were manually verified by examining the aligned reads in the *.bam file using the Integrated Genome Viewer (IGV; Thorvaldsdóttir et al., 2012).

#### 2.4.2 AlkAnilineSeq

Analysis of m^7^G and D modifications in *B. henselae* tRNAs was done by AlkAnilineSeq protocol (Marchand et al., 2018, 2021). This method also detects m^3^C and ho^5^C, but these residues have not been reported in bacterial tRNAs. Eventual cleavage signals may be also observed for s^2^C and ho^5^U intermediates at position 34 of tRNAs. This method exploits sensitivity of certain RNA modified bases to ring-opening at high temperature and alkaline conditions, the resulting damaged base or RNA a basic site is further cleaved by aniline and adapter is ligated to the released 5′-P termini.

#### 2.4.3 HydraPsiSeq

For the mapping of pseudouridine (Psi) modifications, we employed a chemical-based protocol using hydrazine cleavage, termed HydraPsiSeq (Motorin et al., 2007; Behm-Ansmant et al., 2011; de Brouwer et al., 2018). This protocol measures resistance of pseudouridines and m^5^U (rT) residues to the hydrazine cleavage. Subsequent aniline treatments convert damaged U residues to cleavages of the polynucleotide chain. Ligation of the sequencing adapter is done as in the AlkAnilineSeq protocol (see above). Of note, k^2^C (lysidine) present in bacterial tRNA^Ile^_CAU_ at the wobble position shows extensive cleavage upon hydrazine treatment and thus can be detected as positive cleavage signal.

#### 2.4.4 RNA bisulfite sequencing

Analysis of eventual m^5^C modifications in *B. henselae* tRNAs was performed with the well-established bisulfite conversion protocol developed by Schaefer et al. (2009). The EZ RNA Methylation Kit (Zymo Research #R5001) was used for RNA bisulfite treatment followed by desulphonation. Bisulfite-converted RNAs were then end-repaired and subjected to NEBNExt Small RNA Library Prep Set for Illumina (NEB, E7330L), according to manufacturer’s recommendations. Sequencing was performed in SR50 mode, with sequencing reads aligned to C → T converted reference sequence for *B. henselae* tRNAs. 3.1.

## 3 Results and discussion

### 3.1 Gathering tRNA gene sets and tRNA modification nucleoside profiles for two *Bartonella* species

**Figure 1:**
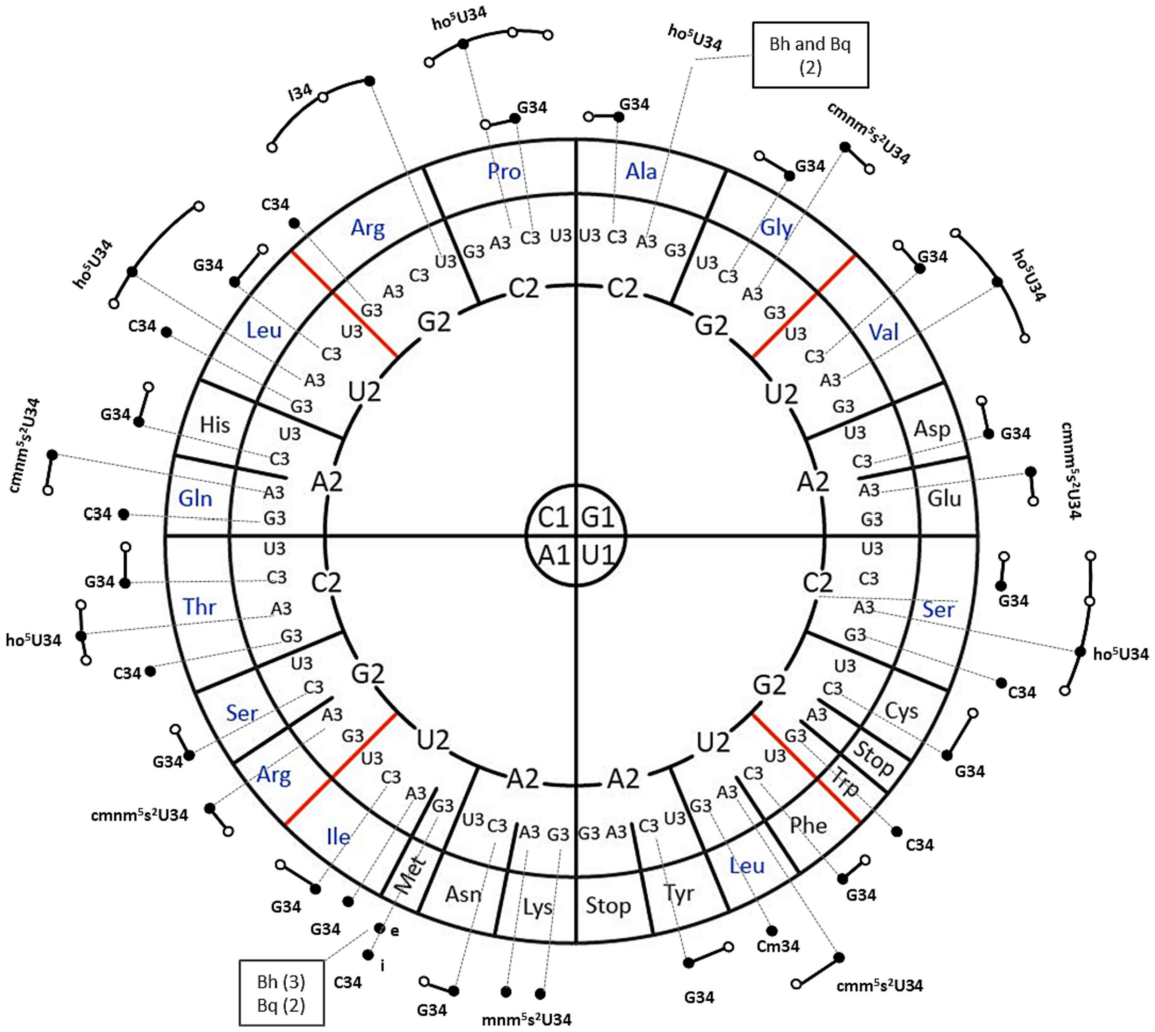
tRNA isoacceptors genes distribution for *Bartonella henselae (Bh) and Bartonella quintana (Bq).* A circular representation of the genetic code, highlighting modified nucleosides, is presented, following the format described by Grosjean and Westhof (2016). In this representation, each amino acid codon sequence is read from the inside out (1-2-3). The dark dots represent the tRNA isoforms encoded by the genome. Additionally, the elongator or initiator tRNA*^Met^*_CAU_ are denoted by “e” and “i,” respectively. The predicted modifications of the ASL of any given isoacceptor *e* and the predicted expanded decoding capacity through wobble or expanded wobble of each isoacceptor are noted by extended dark lines and white circles. All isoacceptors are present in one copy with two exceptions noted in boxes with the numbers of copies in parentheses.

The *B. henselae* genome encodes a total of 43 predicted tRNA genes, specifying 38 distinct iso-acceptors with only two tRNAs with multiple copies (Figure 1). In comparison, the genome of *B. quintana* comprises 42 tRNA genes with an equivalent number of iso-acceptors, albeit with the loss of one of the triplicate copies of initiator-tRNA^Met^_CAU_ (Figure 1). This reduction in iso-acceptors is modest when compared to *E. coli*, with only four losses: tRNA^Arg^_CCT_, tRNA^Gly^_CCC_, tRNA^Pro^_CGG_, and tRNA^SelCys^_UCA_. This contrasts sharply with organisms with reduced genomes, such as mollicutes, which can pare down the number of tRNAs to 28 (Grosjean et al., 2014). Notably, while the total number of tRNA genes in *E. coli* is 86, often present in five copies (Chan and Lowe, 2016), the two *Bartonella* species have significantly reduced the duplicate gene copies of any given iso-acceptor (Figure 1).

In the absence of post-transcriptional modifications, these sets of tRNAs would be incapable of decoding the 64 codons (Grosjean et al., 2010). To illustrate, tRNA^Ile^_CAU_ requires the modification of cytosine to lysidine to decode AUA codons. However, the modification status of tRNA molecules from *Bartonellaceae* was unknown, as none had been previously sequenced (Boccaletto et al., 2018). Consequently, we undertook an LC–MS/MS analysis to search for known tRNA modifications, examining the ribonucleosides obtained from the enzymatic digestion of bulk tRNA extracted from both *Bartonella* species cultivated on HIBB plates, as detailed in the methods section. Here we used two different LC-MS/MS methods (Supplementary Tables S2, S4) to discover modified ribonucleoside in different biological replicate tRNA samples from *B. henselae* and from the more challenging-to-culture *B. quintana* (Supplementary Tables S3, S5). In all, 31 modified ribonucleosides were detected in these samples, 30 with standards and 1 (ms^2^t^6^A) without but with genetic evidence as discussed in the section below (Table 1; Supplementary Tables S3, S5). However, not all modified ribonucleosides in this list are derived from *Bartonella* tRNAs. Indeed, rRNA modifications are routinely detected in analyses of tRNA samples as we previously discussed when analyzing tRNA modification profiles in *Bacillus subtilis* (de Crécy-Lagard et al., 2020) and in the current analysis we can also detect modifications both from rRNA or tRNA from the sheep cells present in the culture media. To predict the tRNA modification profiles of the *Bartonella* species specifically, we combined the results of LC–MS with bioinformatic analyses and NGS-based modification detection methods as described in the next sections.

### 3.2 Predicting the *Bartonella henselae* tRNA modification gene set

**Figure 2:**
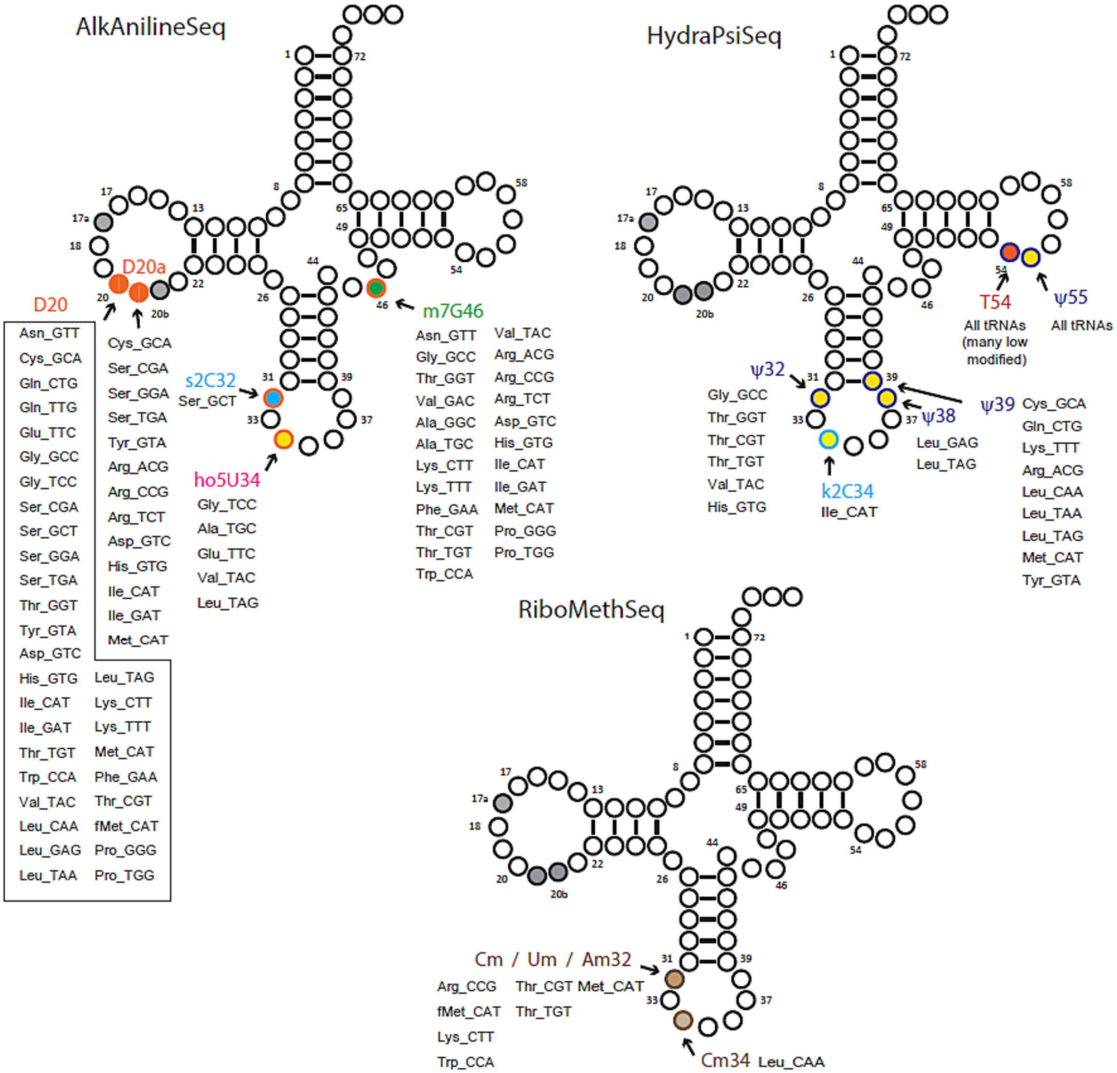
Overview of tRNA modifications mapping by deep sequencing-based protocols: AlkAnilineSeq, HydraPsiSeq and RiboMethSeq. Positions and identities of modified residues found in *Bartonella henselae* tRNA species (shown by amino acid specificity and anticodon) are shown on a canonical cloverleaf tRNA structure. Long tRNA variable loops are not shown.

**Figure 3:**
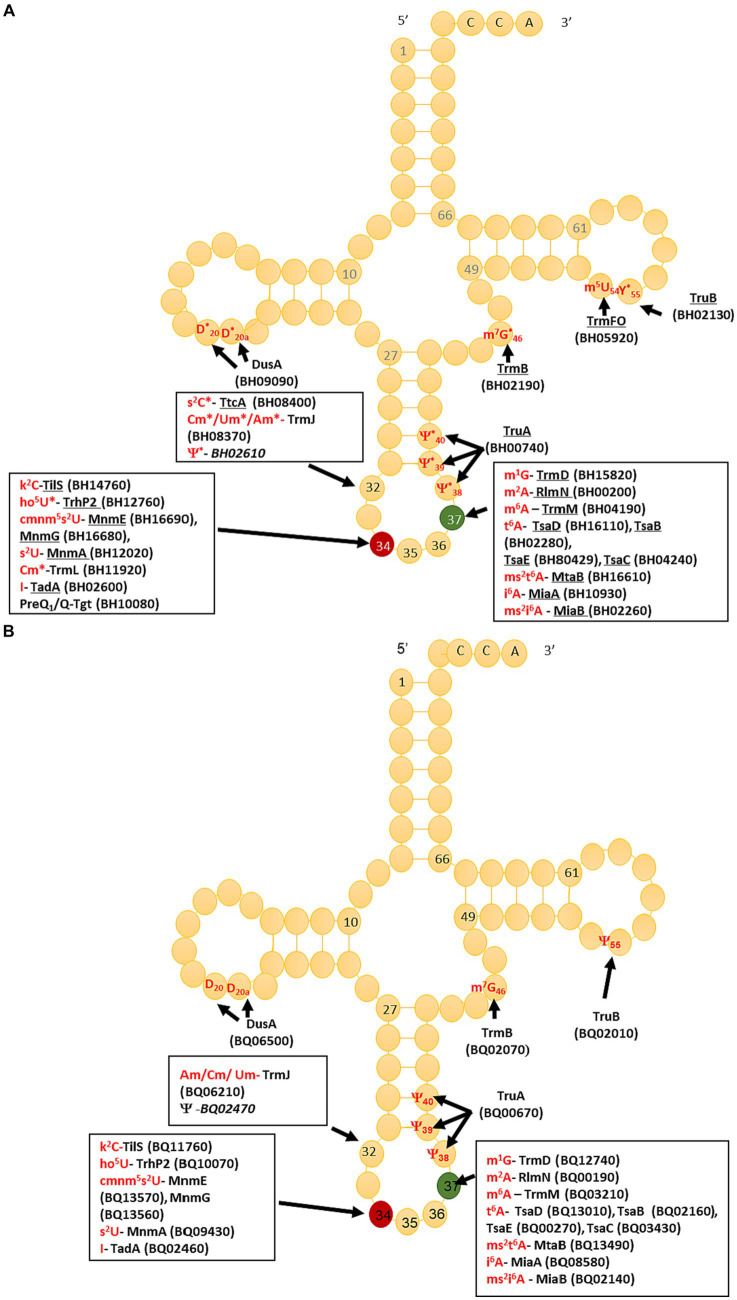
Prediction of *Bartonella henselae* and *Bartonella quintana* tRNA modifications maps and corresponding synthesis proteins. **(A)** *Bartonella henselae*, underlined protein names denote high confidence based on orthology; in red are modifications detected by LC-MS/MS in all samples. A “*” denotes that the modification at this position was confirmed by NGS. Locus tags are given in parentheses, and when italicized, denote a low-confidence prediction. **(B)** *Bartonella quintana*, locus tags are given in parenthesis, and when italicized it denotes a low confidence.

The compilation of tRNA modification genes, initially established for model organisms *E. coli* and *B. subtilis* (de Crécy-Lagard et al., 2020), underwent an update by incorporating two recently identified genes in *B. subtilis* (Jaroch et al., 2023) into the dataset (Supplementary Tables S6, S7). This updated compilation served as a foundation for identifying tRNA modification genes encoded by *B. henselae* strain Houston 1, utilizing both BlastP (Altschul et al., 1997) and advanced search tools provided by BV-BRC (Olson et al., 2023) and UniProt (Bateman et al., 2023). The predictions generated were then cross-referenced with the modifications detected through LC-MS, and with the sequencing-based (RiboMethSeq, AlkAnilineSeq, and HydraPsiSeq) detection analyses results (Figure 2), leading to the creation of the predicted tRNA modification map depicted in Figure 3. In total, we predicted 26 genes participating in the insertion of 23 modifications (Figure 3; Supplementary Table S8).

For 18 of these modifications, the combination of gene information and LC–MS/MS data yielded predictions with high confidence, as highlighted in Figure 3 and Table 1. The position of a few of those such as m^7^G46, ho^5^U34, and s^2^C32 were confirmed by sequencing (Figure 2; Supplementary Figure S1). Additional mapping information was required for the remaining 5 modifications. Certain modification enzymes, like methyltransferases, pseudouridine synthases (Hamma and Ferre-D’Amare, 2006) or dihydrouridine synthases (Bou-Nader et al., 2018), have undergone multiple duplications and shifts in substrate specificity or may exhibit multisite specificity (Barraud and Tisné, 2019). Consequently, predicting their specificities based solely on orthologous relationships can be challenging. Many bacteria possess multiple D modifications, introduced by different enzymes such as DusA/B/C in *E. coli* (Bishop et al., 2002) or a single enzyme like DusB in *M. capricolum* (Faivre et al., 2021). In the *B. henselae* genome, the sole encoded member of the Dus family is a DusA homolog. While *E. coli*’s DusA modifies positions 20/20a (Bishop et al., 2002), we could not exclude the possibility that in *B. henselae*, the enzyme had evolved a relatively broader specificity. However, AlkAnilineSeq analysis conclusively confirmed that the only positions modified by D in *Bartonella* tRNAs were 20/20a (and possibly 20b; Figure 2; Supplementary Figure S1).

In the mapping of Ψ residues, the identification of homologs of TruA and TruB, and the absence of TruC and TruD, suggested modifications at positions 38–40 (TruA) and 55 (TruB) but excluding positions 13 and 65 formed by TruD and TruC, respectively. However, a conclusive determination for position 32, known to be modified in *E. coli* by a dual-specific RluA enzyme that modifies both rRNAs and tRNAs, was not as straightforward. Three predicted rRNA pseudouridine synthases are encoded in the *B. henselae* genome (BH02610, RluC/BH10200, or RluD/BH03820), and any of these might have shifted specificity to modify tRNAs, akin to the observed phenomenon with RluA and RluF in *E. coli* (Addepalli and Limbach, 2016). HydraPsiSeq analysis confirmed the absence of Ψ residues at positions 13 and 65 and the presence of Ψ at position 55 in many tRNAs and at positions 38/39/40 in a few tRNAs (Figure 2; Supplementary Figure S2) confirming the predictions based on the presence/absence of the corresponding synthesis genes. Ψ was also detected at position 32 in several tRNAs (Figure 2; Supplementary Figure S2). We propose that BH02610, the lone orphan pseudouridine synthase, catalyzes the formation of Ψ32 in *B. henselae*, but further experimental validation will be required. HydraPsiSeq protocol also detects 5′-modified U (namely m^5^U), which also shows protection against hydrazine cleavage, and k^2^C (lysidine) which is efficiently cleaved under conditions used in HydraPsiSeq (Supplementary Figure S2) The results of this mapping indicated that U54 in many tRNAs is only partially protected, indicating sub-stoichiometric modification by TrmFO. As anticipated, lysidine-related signal in HydraPsiSeq was found in tRNA^Ile^_CAU_ at wobble position C34 (Supplementary Figure S2). The presence of homologs of TrmJ and TrmL strongly predicts the presence of ribose −2′-O-methylations at positions 32 and 34, respectively, but the exact nature of the modified nucleosides could vary. RiboMethSeq analyses were able to map Am/Cm/Um at positions 32, but only Cm at position 34 (Figure 2; Supplementary Figure S3). Finally, the presence of a homolog of Tgt, the signature enzyme for the queuosine pathway in *B. henselae*, suggested the presence of Q in this organism. However, we previously showed that the Q observed in the LC-MS/MS analysis could be a contamination from mammalian host and that the preQ_1_ precursor could be the biological form of the deazapurine found in this organism (Quaiyum et al., 2023).

A significant number of modifications identified by LC–MS/MS could not be attributed to specific genes (Table 1), including those present in substantial quantities like m^5^C (Supplementary Tables S3, S5). For m^5^C it was confirmed by bisulfite sequencing that indeed it is not present in the *B. henselae* tRNAs (data not shown). The levels of many of these “orphan” modifications exhibited considerable variation among the analyzed samples. For instance, m^2^_2_G was present in certain samples and absent in others (Supplementary Tables S3, S5). One plausible source for these modified nucleosides is rRNA from the bacteria or tRNA/rRNA from the host. Indeed, m^3^U, m^5^C, and m^6^_2_A are well-known rRNA modifications (Boccaletto et al., 2018), while contamination by mammalian host tRNAs, might contribute to the observed pools of m^1^A, G_m_, m^3^C, m^2^_2_G and m^2^G (Boccaletto et al., 2018).

### 3.3 Evolutionary streamlining: decoding capacity maintenance in *Bartonella henselae* through modification complexity reduction

*Bartonella henselae* exhibits a streamlined tRNA modification machinery, evident both in the reduced number of modifications and the simplicity of the existing ones. In comparison to *E. coli*, *B. henselae* encodes less than half the modification genes (26 genes compared to *E. coli*’s 59). Studies on insect symbionts have indicated that modifications of the tRNA body are the first to diminish and can even be entirely lost by reductive evolution, as observed in the genome of louse symbionts (de Crécy-Lagard et al., 2012). Notably, common tRNA body modifications such as D16/17, Ψ13/65, and s^4^U8 are absent in *B. henselae* tRNAs based on the absence of the genes and confirmed by the lack of the NGS signals (data not shown). DusB family is predicted to be the ancestral Dus enzyme (Bou-Nader et al., 2018), and depending on the organism it modifies position 16 or is multisite-specific (Faivre et al., 2021). It seems to have been lost near the root of the *Bartonellaceae* clade (Supplementary Figure S4B). The only remaining Dus enzyme in *B. henselae* is DusA which is found in all *Hyphomicrobiales* (Supplementary Figure S4A). TruC and TruD and ThiI are totally absent in *Alphaprotebacteria*, hence the observed losses in the *Bartonellaceae* are not recent events (Supplementary Figure S5).

Losing modifications in the Anticodon Stem Loop (ASL) should significantly impact decoding capacity, efficiency, and accuracy. Only two ASL modifications found in *E. coli* are absent in *B. henselae*: ac^4^C34 and Ψ35. The first is found in the elongator tRNA^Met^_CAU_ and prevents misreading of the near cognate AUA (Ile) codon (Kawai et al., 1989). The second affects the decoding efficiency of Tyr codon stretches (Addepalli and Limbach, 2016). However, as the synthesis enzymes for both these modifications (TmcA and RluF) seem to be absent in nearly all *Alphaproteobacteria* (Supplementary Figure S5), organisms must compensate for the absence of these modifications.

Interestingly, several complex modifications present in *E. coli* exist in *B. henselae* but in a simpler form that requires a truncated synthesis pathway with fewer genes. The simplification of the Q pathway to salvaging and inserting preQ_1_ and possibly queuine (q) bases was reported previously (Quaiyum et al., 2023), but it looks like all complex pathways have been simplified in this organism. For instance, the cmnm^5^s^2^U precursor, synthesized by MmmGEA enzymes, is present, but its derivative mnm^5^s^2^U, requiring two additional enzymatic steps, is not. Similarly, the ho^5^U precursor is present, but not its derivative, cmo^5^U. Likewise, the t^6^A modification is present but it is not further modified to m^6^t^6^A. These simplifications of complex ASL modifications to minimal forms have facilitated the reduction of the number of tRNA modification genes without affecting the capacity of the 43 *B. henselae* tRNAs to decode the full set of codons (Figure 1). There is only one example of a modification that is more complex in *B. henselae* than in *E. coli*: ms^2^t^6^A. The enzyme involved in its synthesis, MtaB, is widespread in *Alphaprotebacteria*, so its presence in *Bartonellaceae* reflects the evolutionary history of the species (Supplementary Figure S5).

### 3.4 Predicting the tRNA modification gene set in *Bartonella quintana* shows further reductions

**Figure 4:**
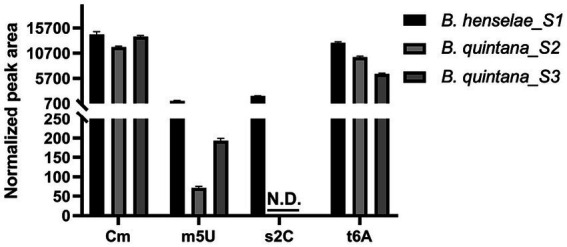
Quantification of Cm, m^5^U, s^2^C, and t^6^A modifications in *Bartonella henselae* and *Bartonella quintana*. LC-MS/MS analysis was performed on 600 ng of small RNA, with signal intensities normalized to the sum of UV absorbances of the canonical ribonucleosides, using an inline UV detector, to correct for differences in amounts of injected RNA. Data represent means ± SD for three technical replicates of one biological replicate for *B. henselae* (S1) and two biological replicates (shown individually) for *B. quintana* (S2, S3). N.D., Not detected.

**Figure 5:**
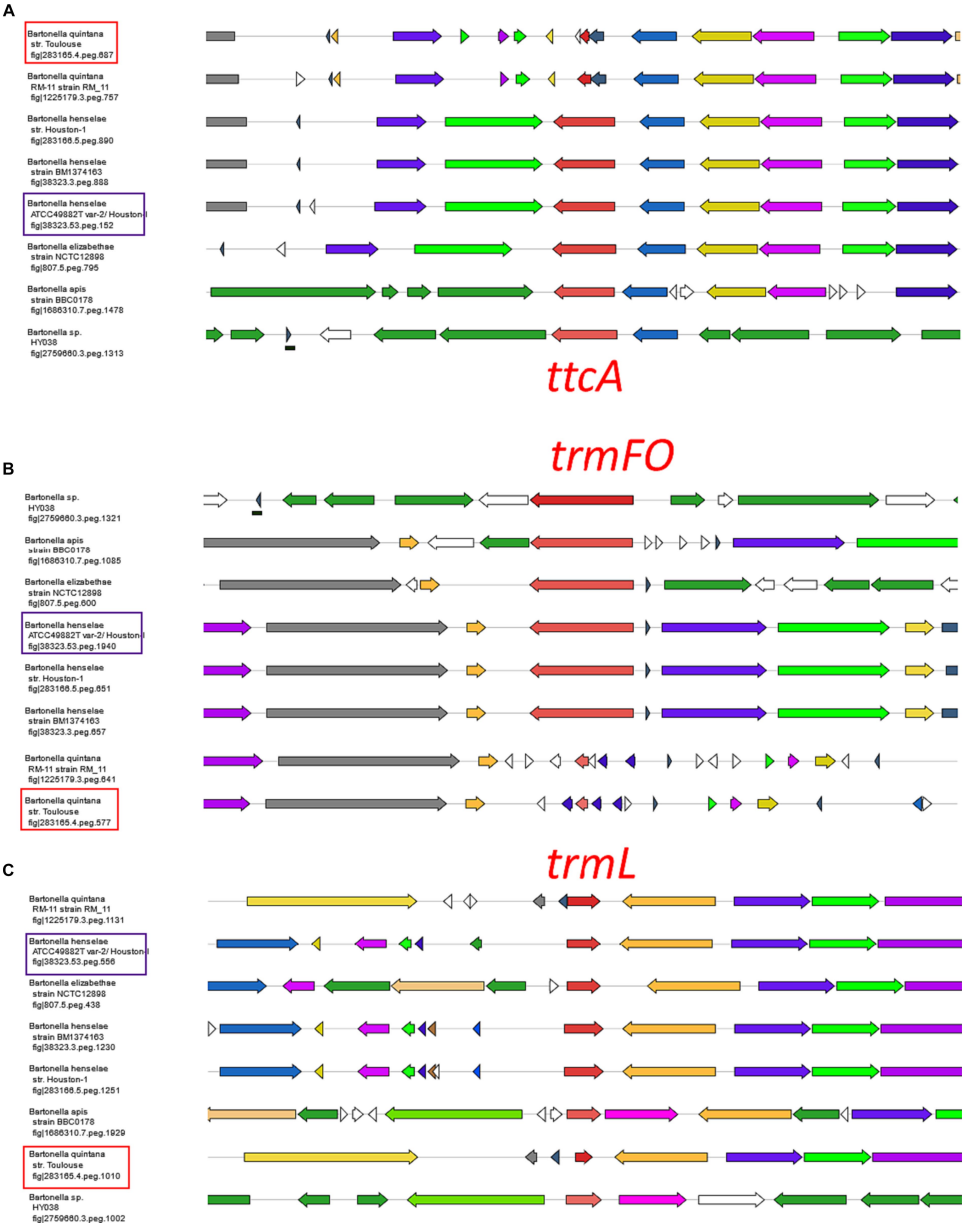
Decay of *ttcA*, *trmL*, and *trmFO genes in Bartonella quintana* species. Gene neighborhoods surrounding the three modification genes in *Bartonella* species. *Bartonella quintana* str. Toulouse and the *Bartonella henselae* Houston-I are boxed in red and purple, respectively. The BV-BRC gene ids (or fig ids) of the specific anchoring genes [*ttcA* in **(A)**, *trmFO* in **(B)** and *trmL* in **(C)**] are given for every cluster.

A parallel analysis was executed to predict the tRNA modification gene sets in *B. quintana*, as depicted in Figure 2B and detailed in Supplementary Table S9. This analysis identified 22 genes linked to 23 modifications. While most genes were found in both *Bartonella* species, instances of gene decay were observed, resulting in the loss of corresponding modifications in the extracted tRNA digests (Supplementary Tables S3, S5; Figure 4). Prior findings had noted the decay of the *tgt* gene (Quaiyum et al., 2023), and three more instances were identified: *ttcA*, *trmFO*, and *trmL*. In each case, the complete gene is present in *B. henselae*, but only fragments are discernible at the corresponding loci in the sequenced *B. quintana* Toulouse genome (Figure 5; Supplementary Table S10). Notably, the decay of *tgt*, *ttcA*, and *trmFO* genes appears consistent across all sequenced *B. quintana* strains, while the truncation of *trm*L seems specific to the *B. quintana* Toulouse strain. Results from LC-MS/MS analyses of bulk tRNAs extracted from *B. quintana* seem to align with these losses. For instance, the disappearance of the *ttcA* gene correlates with the absence of s^2^C in the nucleoside analysis profile from *B. quintana*, contrasting with its presence in *B. henselae* (Figure 4; Supplementary Tables S3, S5). Levels of C_m_ are not dramatically affected in *B. quintana* (Figure 4; Supplementary Tables S3, S5). Given that C_m_ can also be derived from contaminating rRNAs or host tRNAs and that RiboMethSeq for *B. quintana* tRNAs was not performed, additional targeted experiments will be essential to validate the absence of this modification at position 34. Members of the TtcA family are present in most *Bartonella* species (Supplementary Figure S4B). s^2^C32 has been shown to have a role in preventing frameshift (Jager et al., 2004) but is also important to restrict the ability of tRNA^Arg^_ICG_ to decode the rare arginine codon CGA (Vangaveti et al., 2020). Losing the Cm methylation in tRNA^Leu^_CAA_ reduces the efficiency of codon-wobble base interaction, as demonstrated in an amber suppressor system (Benítez-Páez et al., 2010). It is therefore expected that a loss of translation accuracy should occur with the loss of both C_m_ and s^2^C modifications in *B. quintana*, a phenomenon often observed in host-restricted organisms (Melnikov et al., 2018).

Finally, m^5^U levels are reduced by approximately 80% in *B. quintana* compared to *B. henselae* (Figure 4; Supplementary Tables S3, S5). The residual m^5^U could be derived from contaminating rRNA and/or host tRNAs (Boccaletto et al., 2018). Two non-orthologous families of methylases can catalyze the formation of m^5^U54 in tRNA: the SAM-dependent TrmA and the FAD-dependent TrmFO. The two enzymes are mutually exclusive, and genomes encode one or the other (Myllykallio et al., 2018). Members of the alphaproteobacterial clade use TrmFO (Supplementary Figure S5), hence its presence in *Bartonellaceae* is to be expected. The loss of m^5^U54 in *B. quintana* is like what is observed in other organisms with minimal genomes (de Crécy-Lagard et al., 2012; Grosjean et al., 2014).

## 4 Conclusion

This study shows that the tRNA modifications profiles and most corresponding genes can be predicted for the facultative intracellular pathogens *B. henselae* and *B. quintana*, but one must be wary of contamination by host tRNAs and rRNA and combine different types of evidence. Also, it is possible that certain modifications may have been missed in the study, e.g., /if novel pathways are present to insert modifications that could not be chemically identified because of a lack of standards. Nevertheless, even with these potential omissions, it is possible to conclude that while *B. henselae* has not greatly reduced the number of tRNA isoacceptors, they are all in single copy. In addition, the number of modified bases in tRNAs has been reduced from 43 in *E. coli* to 28 in *B. henselae* (a~35% relative reduction). Finally, the number of tRNA modification genes has undergone an even greater reduction with 59 genes in *E. coli* to 26 in *B. henselae* (a~56% relative reduction) because of the simplification of the most complex pathways. Further simplifications of the tRNA modifications apparatus are observed in *B. quintana* with an additional loss of four modifications (preQ_1_, C_m_. s^2^C and m^5^U) compared to *B. henselae*, which correlates with the extensive genome reduction following its divergence from *B. henselae* and specialization for the human host and louse vector (Alsmark et al., 2004). It is also possible that an increase error rate caused by the loss of tRNA modifications in these organisms could lead to a better evasion of the immune system by providing greater variability to surface epitopes, as postulated for other pathogens (Melnikov et al., 2018).

## Data availability statement

The data presented in the study are deposited in the Short Read Archive with the accession number PRJEB72223 and in the PRIDE database with the accession number PXD048805.

## Author contributions

SQ: Investigation, Writing – original draft. JS: Data curation, Investigation, Writing – original draft. VM: Investigation, Methodology, Writing – review & editing. GS: Data curation, Investigation, Methodology, Visualization, Writing – original draft, Writing – review & editing. CR: Data curation, Investigation, Visualization, Writing – review & editing. YM: Funding acquisition, Methodology, Supervision, Visualization, Writing – original draft, Writing – review & editing. PD: Funding acquisition, Project administration, Supervision, Writing – review & editing. MM: Conceptualization, Investigation, Methodology, Writing – review & editing. VC-L: Conceptualization, Data curation, Funding acquisition, Investigation, Project administration, Resources, Supervision, Visualization, Writing – original draft, Writing – review & editing.

## Footnotes

^1^ http://gtrnadb.ucsc.edu/GtRNAdb2/genomes/bacteria/Bart_hens_Houston_1/

^2^ http://gtrnadb.ucsc.edu/GtRNAdb2/genomes/bacteria/Bart_quin_Toulouse/

^3^ https://phylot.biobyte.de

^4^ http://www.gizmogene.com/

^5^ https://itol.embl.de

^6^ https://software.broadinstitute.org/morpheus

^7^ http://www.ebi.ac.uk/pride

^8^ https://www.ncbi.nlm.nih.gov/sra/
